# Effects of β2-Adrenergic Receptor Gene Polymorphisms on Ritodrine Therapy in Pregnant Women with Preterm Labor: Prospective Follow-Up Study

**DOI:** 10.3390/ijms150712885

**Published:** 2014-07-21

**Authors:** Jin Young Park, Na Ra Lee, Kyung Eun Lee, Sunny Park, Young Ju Kim, Hye Sun Gwak

**Affiliations:** 1 College of Pharmacy and Division of Life and Pharmaceutical Sciences, Ewha Womans University, Seoul 120-750, Korea; E-Mails: kellyjeans87@naver.com (J.Y.P.); narara87@hanmail.net (N.R.L.); kaylee@cbnu.ac.kr (K.E.L.); psunny0708@naver.com (S.P.); 2 College of Pharmacy, Chungbuk National University, Cheongju, Chungbuk 361-763, Korea; 3 Department of Obstetrics and Gynecology, Ewha Womans University School of Medicine, Seoul 158-710, Korea

**Keywords:** ritodrine, preterm labor, *β2-adrenergic receptor* gene, single nucleotide polymorphism

## Abstract

This study aimed to evaluate the effects of *β2-adrenergic receptor* (*ADRB2*) gene polymorphisms on ritodrine therapy outcomes in patients with preterm labor. Genotyping analysis of *ADRB2* gene (rs1042713, rs1042714, rs1042717, rs1042718, and rs1042719) was performed on 137 patients with preterm labor. Survival analysis was conducted for the effects of SNPs on the median time to delivery as a primary outcome. The median time to delivery in the study patients was 349.3 h. Gestational age at admission and modified Bishop scores revealed significant effects on time to delivery (*p* < 0.001). Among studied SNPs, rs1042717 and rs1042718 showed linkage disequilibrium in this population, and their effects on time to delivery were marginally significant (*p* < 0.1). Patients with variant-homozygotes in the rs1042713 showed considerably shortened time to delivery compared to wild-allele carriers. The rs1042719 polymorphism significantly affected time to delivery in both univariate and multivariate analysis; the GC and CC carriers showed 64% decrease in time to delivery compared to the wild-type homozygote carriers. Based on the results, it was concluded that the gene polymorphisms of *ADRB2* could affect ritodrine therapy in patients with preterm labor. However, given the single-center and the relatively small sample size, our hypothesis requires further independent validation using multi-center and large sample size.

## 1. Introduction

Preterm birth is defined as spontaneous labor occurring before 37 completed weeks of gestation. It is the leading cause of neonatal mortality and morbidity in newborns without congenital anomalies or chromosomal abnormalities [1,2]. According to 2012 statistics, the rate of preterm birth ranges between 5% and 18% of babies born across the world [3]. Preterm birth also has major socioeconomic implications with associated hospital stays [4]. Tocolytic drugs with a variety of mechanisms are being used to relax the uterus and suppress the uterine contractions to prevent preterm birth.

Ritodrine is a β2-adrenergic receptor (ADRB2) agonist, resulting in uterine smooth muscle relaxation. Although it has been widely used in several European and Asian countries, the efficacy of ritodrine was not consistent [4,5]. Numerous factors are believed to contribute to the speed and outcome of delivery, but several studies implicated the importance of association between genetic predisposition and preterm birth [6].

Human *ADRB2* gene is encoded on chromosome 5, and its genetic variability has been widely characterized with several single nucleotide sequence variations [7]. Especially, missense mutations encoding for amino acids 16 and 27 of the extracellular *N*-terminus of the β2-adrenergic receptor have been studied in many areas including β-blocker therapy in cardiovascular disorders and β2-agonist treatment in respiratory disorders [8,9,10,11,12]. However, not many studies have been conducted in the preterm labor treatment with β2-agonists, such as ritodrine. Since it is important to detect a correlation between drug phenotypes and single nucleotide polymorphisms (SNPs) studied, in order to utilize this correlation to benefit patients, the purpose of this study was therefore to evaluate the association between efficacy of ritodrine and *ADRB2* gene polymorphisms in patients with preterm labor.

## 2. Results

### 2.1. Effects of Patient Characteristics on Time to Delivery

One hundred and fifty-four patients were enrolled, and electronic medical records of the patients were reviewed in Ewha Womans University Mokdong Hospital. Five patients who used ritodrine as a premedication for McDonald operation and eight patients who already had severe symptoms that led to preterm labor before hospital admission were excluded. Four patients who had to give up their babies because of the critical danger were excluded. Total seventeen patients were excluded by exclusion criteria, and one-hundred-thirty-seven patients were included. The mean maternal and gestational ages at admission were 31.6 ± 4.7 years and 29.5 ± 3.8 weeks, respectively. Seven patients had multiple gestations among 137 studied patients. The mean cervical dilation was 1.3 ± 1.2 cm.

The effects of demographic and clinical characteristics of patients on time to delivery are displayed in Table 1. From univariate analysis, gestational ages at admission and Modified Bishop scores were statistically significant factors for time to delivery in preterm labor patients with ritodrine therapy (*p* < 0.001).

**Table 1:** Effects of demographic characteristics on time to delivery.

Characteristic	Number	Time to Delivery Median (95% CI)	*p* Value
Age			0.582
<35 years	109	772.0 (388.58–1155.42)	
≥35 years	28	491.5 (5.47–977.47)	
Gestational age at admission			<0.001
<32 weeks	87	1344.0 (1033.17–1654.83)	
≥32 weeks	50	231.0 (112.66–349.35)	
Modified Bishop score *			<0.001
<3	78	978.5 (386.48–1570.52)	
≥3	29	161.0 (32.46–289.54)	
Mode of delivery			0.567
Normal delivery	58	614.0 (325.33–902.67)	
Cesarean-section	64	539.9 (0.00–1229.55)	

* Modified Bishop score is the sum of dilatation score and effacement score. Dilatation score: 0, <l cm; 1, 1 to 3 cm; 2, 3 to 5 cm; 3, ≥5 cm. Effacement score: 0, 0% to 40%; 1, 40% to 60%; 2, 60% to 80%; 3, ≥80%.

### 2.2. Effects of Genotypes on Time to Delivery

The genotypes and allele frequencies of studied SNPs of *ADRB2* gene are shown in Table 2. Minor allele frequencies ranged between 9.7% and 49.6%. The observed frequencies were consistent with Hardy–Weinberg equilibrium in all SNPs. The median time to delivery was 967.6, 741.0, and 287.1 h in patients with the wild-type homozygotes, heterozygotes, and mutant-type homozygotes in the Arg16Gly polymorphism, respectively. On the contrary, in the Gln27Glu polymorphism, longer time to delivery was found in patients with variant allele compared to those with the wild-type homozygotes. However, there was no significant association between the above two SNPs and pharmacologic responses of ritodrine treatment.

**Figure 1:**
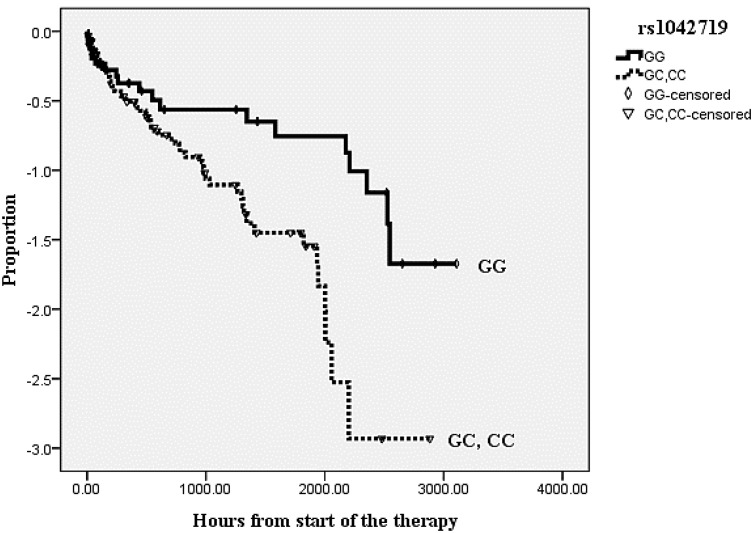
Survival curve comparing GG group with the other two genotypes (GC, CC group, *p* = 0.019) of *ADRB2* rs1042719 by Kaplan–Meier survival analysis.

There was a linkage disequilibrium between c.252 G>A and c.523 C>A (*r*^2^ = 0.984), and their effects on time to delivery were marginally significant (*p* < 0.1). Patients with wild-homozygotes showed prolonged time to delivery compared to those with variant alleles. The c.1053 G>C polymorphism significantly affected time to delivery; it took much shorter time to delivery in preterm labor patients with the GC, CC genotype than those with the GG genotype (*p* = 0.019, Figure 1). Cox’s proportional-hazards analysis demonstrated that maternal ages, modified Bishop scores, gestation ages at admission, and rs1042719 were significant predictors of time to delivery. Especially, the GC and CC carriers showed 64% decrease in time to delivery compared with the GG genotype patients in rs1042719 (*p* = 0.020) after adjusting other variables (maternal age and variables, which showed *p* < 0.1 from the univariate analysis) (Table 3).

**Table 2:** Effects of grouped genotypes in β-2 adrenergic receptor on the time to delivery.

Gene Polymorphism	Minor Allele Frequency (%)	Grouped Genotype	Number of Patients	Time to Delivery (h) Median (95% CI)	*p* Value
*ADRB2* rs1042713	49.6%	AA, AG	101	828.0 (472.39–1183.61)	0.089
(c.46 A>G, Arg16Gly)		GG	34	287.1 (28.35–545.91)	
*ADRB2* rs1042714	9.7%	CC	109	684.5 (415.17–953.83)	0.932
(c.79 C>G, Gln27Glu)		CG, GG	25	1030.3 (0.00–2109.88)	
*ADRB2* rs1042717	39.2%	GG	48	1319.0 (330.50–2307.50)	0.098
(c.252 G>A, Leu84Leu)		GA, AA	86	566.0 (304.13–827.87)	
*ADRB2* rs1042718	39.8%	CC	48	1319 (199.56–2438.44)	0.061
(c.523 C>A, Arg175Arg)		CA, AA	89	539.9 (330.39–749.45)	
*ADRB2* rs1042719	47.4%	GG	35	1584.0 (0.00–3597.95)	0.019
(c.1053 G>C, Gly351Gly)		GC, CC	102	566.0 (314.53–817.47)	

There were a total of 8 failures in genotyping 5 SNPs of 137 patients.

**Table 3:** Multivariate analysis for the time to delivery.

Factor	Odds Ratio (95% CI)	*p* Value
Age (≥35 years)	0.51 (0.28–0.93)	0.027
Modified Bishop score (≥3)	0.31 (0.18–0.55)	<0.00
Gestational age at admission (≥32 weeks)	0.25 (0.14–0.46)	<0.00
*ADRB2* rs1042719	0.36 (0.15–0.85)	0.020

Heterozygote and mutant homozygote *vs.* wild-type homozygotes; Adjusted for all factors of which *p*-value was less than 0.1 from univariate analysis.

### 2.3. Effects of Genotypes on Proportions of Patients Who Remained Undelivered

Proportions of patients who remained undelivered at 24 h, 48 h, and 10 days were not significantly different among the studied genotypes except for the Arg16Gly at 10 days; those with the Arg allele had approximately 1.6 times greater proportion compared to those with the variant-type homozygotes (Table 4).

**Table 4:** Effect of genotypes of β-2 adrenergic receptor on percentages of patients who remained undelivered.

Gene Polymorphism	Genotype	24 h		48 h		10 day	
		Proportion (%)	*p* Value	Proportion (%)	*p* Value	Proportion (%)	*p* Value
*ADRB2* rs1042713	AA, AG	54/62 (87.1)	0.860	50/62 (80.6)	0.693	39/62 (62.9)	0.035
	GG	23/26 (88.5)		20/26 (76.9)		10/26 (38.5)	
*ADRB2* rs1042714	CC	69/79 (87.3)	0.359	64/79 (81.0)	0.819	42/74 (56.8)	0.807
	CG, GG	18/19 (94.7)		15/18 (83.3)		8/15 (53.3)	
*ADRB2* rs1042717	GG, GA	73/83 (88.0)	0.499	65/82 (79.3)	0.171	43/74 (58.1)	0.295
	AA	15/16 (93.8)		15/16 (93.8)		7/16 (43.8)	
*ADRB2* rs1042718	CC, CA	74/84 (88.1)	0.508	66/83 (79.5)	0.177	44/75 (58.7)	0.275
	AA	15/16 (93.8)		15/16 (93.8)		7/16 (43.8)	
*ADRB2* rs1042719	GG, GC	70/79 (88.6)	0.808	63/78 (80.8)	0.602	42/70 (60.0)	0.165
	CC	19/21 (90.5)		18/21 (85.7)		9/21 (42.9)	

## 3. Discussion

Genetic contributions to preterm birth have been studied, recently focusing on genomic and proteomic approaches to diagnose and determine the mechanism of preterm labor. The genes or proteins investigated are involved in inflammatory reactivity or uterine contractility [13,14]. *ADRB2*, which is expressed in smooth muscle cells of the myometrium, inhibits uterine contraction when it is activated. The association between *ADRB2* polymorphisms and preterm labor has been focused on codons 16 and 27. The results were not consistent depending on the outcomes measured and study populations and did not favor a major impact of *ADRB2* polymorphisms at positions 16 and 27 to the risk of preterm labor [15,16,17].

Studies of genetic polymorphisms suggest that it may account for 15%–30% of the variation in drug response [18]. This study tried to approach from an aspect of different responses of tocolytic drugs due to genetic variances. Ritodrine binds to β2 adrenergic receptors on outer membrane of myometrial cell, activating adenylyl cyclase to increase the level of cAMP, which decreases intracellular calcium and leads to uterine contraction reduction [19]. Genetic variation in the structure of *ADRB2* is a potentially significant source of variability in the response to β2-agonist drugs. Most commonly studied SNPs of *ADRB2* gene are Arg16Gly (c.46 G>A) and Gln27Glu (c.79 C>G), non-synonymous variants, resulting in amino acid substitutions. The frequencies of the Gly16 and Glu27 allele were 49.6% and 9.7% in this study, which were consistent with the previous study [20].

In terms of the effects of β2-agonist on preterm labor, the clinical outcomes after hexoprenaline administration to white women diagnosed with preterm labor were studied *ADRB2* gene polymorphisms of Arg16Gly and Gln27Glu, which are two among five SNPs that we studied [21]. The results showed that pregnant women with Arg16 had a strong trend toward greater pregnancy prolongation, compared to the other genotype groups. The result was consistent with our results; patients with Arg16 alleles showed relatively prolonged time to delivery compared to those with the other genotype. Although, both studies narrowly missed to achieve statistical significance (*p* = 0.058 and 0.089, respectively).

In this study, patients with Arg16 alleles showed relatively prolonged time to delivery compared to those with the other genotype. Although Arg16 alleles did not show significant prolongation of time to delivery, significantly greater proportions of patients who remained undelivered at 10 days were found in the Arg16 allele carriers compared to those with Gly16 allele homozygotes. This result was attributable to not only the protecting effect of the Arg16 allele but also the receptor downregulating effect of Gly16 allele. An *in vitro* study showed that the Gly16 allele resulted in enhanced agonist-promoted down-regulation. The *in vitro* study also reported that the Glu27 allele was resistant to receptor down-regulation [22]. However, significant association between the Gln27Glu polymorphism and time to delivery in preterm labor patients with ritodrine therapy was not found in this study.

Gln27Glu is in high linkage disequilibrium with Arg16Gly [23]. Therefore, Gln27Glu could be considered as a cofactor of Arg16Gly effects. To obtain complementary information regarding the effectiveness of ritodrine therapy, the haplotype analysis of the two genotypes was carried out. Although patients with both Gly16 and Gln27 homozygotes (median time: 215.7 h) showed shorter time to delivery than those with the other haplotypes (median time: 828.0 h), significant association was not found (*p* = 0.285).

Strong linkage disequilibrium between rs1042717 and rs1042718 was found in this study, consistent with earlier studies [24,25]. Significantly longer time to delivery in GG allele carrier of rs1042719 was obtained after adjusting other covariates. Unlike rs1042713 and 1042714, rs1042717, 1042718, and 1042719 are synonymous polymorphisms, which were not expected to change the β2-adrenoreceptor activity. The effect of synonymous SNP in the *ADRB2* gene on the ritodrine therapy in this study was possibly attributable to the linkage disequilibrium with other SNPs, which have functional activities [26]. In addition, a recent study showed that synonymous coding SNPs can affect the translation rate of mRNA, resulting in the change of protein amount produced and the post-translational modification of the protein [27]. Another possibility is that it interferes with an exon-splicing enhancer to affect RNA processing [28].

Although SNP detection and genotyping studies have shown to explain and diagnose many diseases and to describe the variation in drug responses, most findings have not been replicated. This is because clinical outcomes are affected by other factors as well as genetic predisposition. Especially, the long-term efficacy and therapeutic usefulness of β2-agonist drugs have been questioned in many studies, and the limitation of the therapeutic value of β2-agonist drugs was attributable to the desensitization of β-adrenergic receptors in the myometrium by agonist-stimulation [29,30]. In the animal study with preterm labor, ritodrine initially reduced labor contractions but the effects decreased within 16 h [31]. The reduced effects were explained by a reduced cAMP response to β2-agonist drugs and by decreased number of β-adrenergic receptors [29,30]. Furthermore, studies showed that neither the Arg16Gly nor the Gln27Glu polymorphism affected the extent of agonist-induced desensitization, but the Glu27 homozygotes exhibited a slower desensitization compared to the other genotypes [32,33].

Another consideration for clinical application of genomic studies is that failure to achieve statistical significance does not necessarily mean clinical insignificance. In this study, most SNPs shortened the time to delivery by more than 50% but statistical significance was not found. This was possibly due to the small sample size, resulting in underpowered study. Given the single-center and the relatively small sample size, our hypothesis requires further independent validation using multi-center and large sample size.

## 4. Materials and Methods

### 4.1. Study Patients

This prospective follow-up study was conducted at Ewha Womans University Mokdong Hospital from January 2010 to February 2013, approved by Ethics Committee of the Ewha Womans University Mokdong Hospital Institutional Review Board. (IRB No.: 217-1-26, 6 January, 2010) Patients were eligible for participation if they met following criteria: preterm labor with intact membrane, gestational age of 20 to 36 weeks, ≥18 years of age, uterine contractions with a frequency of 3 per 10 min with cervical change, and provision of written informed consent. Exclusion criteria were severe pre-eclampsia, placenta abruptio, fetal distress, severe oligohydroamnios, fetal/placental/ amniotic abnormalities, placenta previa, severe spontaneous premature rupture of membrane, and women whose continuation of pregnancy would be dangerous for them. Patients treated with tocolytics other than ritodrine were excluded. In addition, cases in which ritodrine was used for preventing uterine contraction during the Mcdonald operation were excluded.

### 4.2. Drug Administration

Ritodrine (Lavopa^®^; JW Pharmaceutical, Seoul, Korea), was given as an intravenous infusion at an initial rate of 0.05 mg/min and increased by 0.05 mg/min every 10 min, until the desirable uterine response was obtained. Intravenous treatment was discontinued during uterine quiescence. Patients who achieved uterine quiescence received maintenance therapy via an infusion of 0.05 mg/min for 12 to 48 h.

### 4.3. Outcomes and Data Collection

The primary end point was time (hour) to delivery. Secondary endpoint was proportion of patients who remained undelivered at 24 h, 48 h, and 10 days. Patients’ data, including maternal age, gestational age at admission, type of gestation, cervical dilation, Modified Bishop score, and mode of delivery, were collected. The paper-based and electronic medical records of patients were reviewed.

### 4.4. Genotyping Methods

Genomic DNAs were extracted from EDTA–blood samples by usage of QIAamp DNA Blood Mini Kits (QIAGEN GmbH, Hilden, Germany) according to standard procedures recommended by a manufacturer. The following SNPs were genotyped of *ADRB2* gene using The TaqMan^®^ allelic discrimination technique by RT-PCR system (ABI 7300, Applied Biosystem, Carlsbad, CA, USA): rs1042713 (c.46 A>G, Arg16Gly), rs1042714 (c.79 C>G, Gln27Glu), rs1042717 (c.252 G>A, Leu84Leu), rs1042718 (c.523 C>A, Arg175Arg), and rs1042719 (c.1053 G>C, Gly351Gly).

### 4.5. Statistical Analysis

Continuous variables were compared by the Student’s *t*-test; when there were more than two comparison groups, a one-way ANOVA was used. If the variables were not normally distributed, as determined by the one-sample Kolmogorov–Smirnov and Levene tests, additional Mann–Whitney and Kruskal–Wallis tests were used. The Chi-square test was used to compare categorical variables. The interval from start of treatment to delivery was analyzed with Kaplan–Meier survival data analysis method (log-rank test). Cox’s proportional-hazards model was used for exploratory multivariate analysis for the interval from start of treatment to delivery. All the analyses were based on two-tail statistics. The data were analyzed using Statistical Package for Social Sciences Version 17.0 for Windows (SPSS 17.0K, SPSS Inc., Chicago, IL, USA). A *p*-value of less than 0.05 was considered statistically significant.

## 5. Conclusions

The polymorphisms of *ADRB2* gene could affect ritodrine therapy in patients with preterm labor. Given the burden of preterm labor, this pharmacogenomic study could have the potential to improve the management of this disorder by accounting for some of the inter-individual variability in pharmacologic response of ritodrine therapy. Since the study population is uni-ethnic, further study is required on other populations.
